# Knowledge, Attitude, Practice, and Associated Factors of First Aid Services Related to Motorcycle Accidents among Motorcycle Drivers in Dilla Town, Southern Ethiopia: A Cross-Sectional Study

**DOI:** 10.4314/ejhs.v35i5.8

**Published:** 2025-09

**Authors:** Mesafint Tilahun Alemayehu, Tola Getachew Bekele, Merahi Kefyalew, Wagari Tuli

**Affiliations:** 1 Department of Nursing, Dilla University Referral Hospital, Dilla, Ethiopia; 2 Department of Nursing, Jimma University Medical Center, Jimma, Ethiopia; 3 Department of Emergency Medicine, College of Health Science, Addis Ababa University, Addis Ababa, Ethiopia

**Keywords:** First aid, motorcycle drivers, knowledge, attitude, practice

## Abstract

**Background:**

First aid is a vital initial step in reducing serious injuries and improving the chances of survival. However, limited literature exists on first aid services among motorcycle drivers in developing countries, including Ethiopia. This study aimed to assess the knowledge, attitude, practice, and associated factors related to first aid services for motorcycle accidents among drivers in Dilla town, Southern Ethiopia.

**Methods:**

A community-based cross-sectional study was conducted from March 15 to April 15, 2023, among randomly selected motorcycle drivers in selected ketenas of Dilla town. Data were entered into EpiData 4.6 and exported to SPSS version 26 for coding, cleaning, and analysis. Binary logistic regression was performed to assess the association between dependent and independent variables, with a p-value of <0.05 considered statistically significant.

**Results:**

A total of 272 respondents participated in the study, with a mean age of 26.21 ± 4.95 years. More than half of the respondents, 149 (54.8%), had good knowledge, and 140 (51.5%) had a positive attitude, while only 109 (40.1%) demonstrated good practice regarding first aid services. After adjusting for confounders, attending college or university (AOR = 3.937; 95% CI: 1.175–13.194) was significantly associated with good knowledge. Being aged ≥33 years (AOR = 4.4; 95% CI: 1.56–12.6) was significantly associated with good practice.

**Conclusion:**

The study found that nearly half of motorcycle drivers in Dilla town had adequate knowledge and positive attitudes toward first aid, but only about two-fifths demonstrated good practice. Higher educational level and older age were significantly associated with good knowledge and practice of first aid, respectively.

## Introduction

First aid is immediate care provided to an individual experiencing sudden illness or injury, with the goal of preserving life, preventing the condition from worsening, and reducing the severity of injuries. Before medical personnel arrive at the scene or transport victims to a hospital, basic first aid can be administered by laypeople (1). The fate of road traffic accident (RTA) victims is greatly influenced by the “golden hour,” the first hour following trauma. In many cases, an individual's condition may deteriorate if first aid is not administered promptly, potentially affecting long-term health and quality of life, or even leading to death (2).

In developing nations, motorcycles serve various purposes, including mobility, transportation, recreation, and economic activities. Because motorcycles are small, fuel-efficient, and easy to maneuver in congested areas, they are becoming increasingly popular (3). However, many motorcycle riders ignore speed limits, lack adequate driving skills, and carry more passengers than recommended. These factors contribute to a rising number of motorcycle accidents and related injuries and fatalities (4).

The risks of riding a motorcycle are high due to the absence of protective structures to safeguard drivers and passengers. Victims of motorcycle accidents often include drivers, passengers, and pedestrians. Motorcycle injuries, though underreported, represent a growing public health problem and contribute substantially to overall RTAs, which are leading causes of disability and death in developing countries (5).

Globally, motorcycles are involved in 23% of RTA-related deaths (6). In low- and middle-income countries, motorcycle-related accidents are among the most frequent causes of traffic-related injuries and fatalities across all age groups (7). In sub-Saharan Africa, motorcycle-related RTAs account for 13% of all traffic fatalities (8). In Ethiopia, motorcycle drivers represent 21.0% of RTAs (7). Regional studies have also shown higher crash rates than national averages. For example, cross-sectional studies in Hawassa, Arbaminch, Wolayita, and Wolayita Zone Kindo Koyisha Woreda reported that motorcycle crashes accounted for 55.1%, 40%, 31.2%, and 65.1% of road traffic injuries, respectively (9-12).

Beyond the morbidity and mortality associated with RTAs, the resulting economic impact is alarming. RTAs impose long-term healthcare needs, increased medical costs, and financial losses on individuals, families, and communities (13). In Ethiopia, RTAs have become a significant public health burden. Injuries occur for diverse reasons and demand a multi-sectoral approach, including measures to improve the quality and accessibility of emergency medical services (14). Typically, RTA victims in Ethiopia are transported to the nearest health facility without receiving professional care at the scene. Transport is usually provided by the involved vehicle, volunteer drivers, or ambulances, if available.

Most studies on knowledge, attitude, and practice (KAP) of first aid among drivers have focused on general populations. Limited studies exist regarding motorcycle drivers, both globally and in Ethiopia. In particular, no studies have explored the KAP of motorcycle drivers toward first aid in Dilla town, where motorcycles are the main means of transportation. Since accidents affect drivers, passengers, and pedestrians alike, assessing the KAP of motorcycle drivers regarding first aid is essential to guide interventions and reduce complications at accident scenes.

Therefore, this study aimed to fill this gap by exploring the first aid knowledge, attitude, and practice of motorcycle drivers in Dilla town, Southern Ethiopia.

## Materials and Methods

**Study area and study period:** The study was conducted in Dilla town, the administrative center of the Gedeo Zone in the Southern Nations, Nationalities, and Peoples' Region (SNNPR). It is located 395 km from the capital city, Addis Ababa, and 94 km from Hawassa. According to the 2007 census, Dilla town had an estimated population of 81,644. The town consists of five kebeles: Chichu, Harowolaabu, Odaya'a, Haroressa, and Sessa. Each kebele is divided into three ketenas, totaling 15 ketenas. According to the Dilla town transport authority, 4,200 motorcycles were registered with license plates at the time of the study. Data collection was carried out from March 15 to April 15, 2023.

**Study design**: A community-based cross-sectional study design was employed on motorcycle drivers in Dilla town.

**Study population**: Sampled motorcycle drivers from the selected ketenas who fulfilled the inclusion criteria.

**Inclusion criteria**: Registered motorcycles with license plates.

**Exclusion criteria**: Drivers absent during the data collection period due to social reasons. Motorcycles without license plates.

**Sample size and sampling procedure**: The sample size was determined using a single population proportion formula. Since no similar study had been conducted in Ethiopia, the researchers assumed p = 50%.

**Sampling procedure**: From the five kebeles, four were randomly selected. Each selected kebele has three ketenas, of which two were chosen using the lottery method. Based on the number of motorcycle drivers in each selected ketena, proportional allocation was applied, and systematic random sampling was used to select participants. The sampling interval (Kth) was 3 (calculated by dividing the source population by the sample size). The first respondent was chosen by lottery, and then every third driver was selected until the sample size was achieved.

While Knowledge, Attitude, Practice were dependent variables; Age, Sex, Marital status, Educational level, Work experience were independent variables.

### Operational definitions

**Knowledge of first aid:** Awareness of first aid components, recognition and management of bleeding, positioning of victims during an MCA, and victim transportation. Respondents scoring at or above the mean were considered to have good knowledge; those scoring below the mean were considered to have poor knowledge.

**Attitude toward first aid:** Willingness to provide first aid and belief in its necessity at accident scenes. Respondents scoring at or above the mean were considered to have a positive attitude; those scoring below the mean were considered to have a negative attitude.

**Practice of first aid:** Actions taken during incidents involving respiratory problems, severe bleeding, or fractures. Respondents scoring at or above the mean were considered to have good practice; those scoring below the mean were considered to have poor practice.

**Data collection tools and techniques**: Data were collected through face-to-face interviews using a structured questionnaire adapted from previous studies (15,16). The tool included sociodemographic characteristics and items to assess knowledge, attitude, and practice of first aid in motorcycle accidents. It was prepared in English, translated into Amharic, and then back-translated into English for consistency. Three BSc nurses served as data collectors under the supervision of one MSc nurse. The principal investigator coordinated the process and explained the study's purpose.

**Data quality control:** A pretest involving 5% of the sample was conducted two weeks before data collection. Data collectors received one day of training on the study objectives, interview techniques, and data collection procedures. Questionnaires were checked daily for completeness, and issues were addressed immediately. Incomplete questionnaires were excluded before data entry.

**Data processing and analysis**: Data were verified, coded, and entered into EpiData version 4.6, then exported to SPSS version 26 for analysis. Categorical variables were summarized using frequencies and percentages. Descriptive statistics such as means and standard deviations were also calculated. Responses were coded as “1” for correct and “0” for incorrect, and mean scores were used as cut-off points to classify knowledge (good/poor), attitude (positive/negative), and practice (good/poor). Binary logistic regression was conducted to examine associations between dependent and independent variables. Variables with p < 0.25 in the univariate analysis were entered into multivariable analysis. In multivariable analysis, variables with p < 0.05 were considered statistically significant and reported with adjusted odds ratios (AOR) and 95% confidence intervals.

## Results

**Sociodemographic characteristics of motorcycle drivers**: In this study, 272 respondents participated out of a total sample of 288, giving a response rate of 94.5%. The mean age of participants was 26.21 years (SD ±4.95). The majority, 261 (96%), were male. Regarding marital status, 138 (50.7%) were single and 134 (49.3%) were married. Most respondents, 166 (61.0%), had 1–5 years of motorcycle driving experience, followed by 55 (20.2%) with 6–10 years of experience. Concerning education, 109 (40.1%) had attended primary school (grades 1–8), while 90 (33.1%) had attended secondary school (grades 9–12), and 55 (20.2%) had attained education above secondary level (Table 1).

**Table 1:** Shows the Sociodemographic characteristics of the motorcycle drivers, Dilla town, southern Ethiopia, 2023

Variable	Response	Frequency	Percent
Age	17-24	102	37.5
	25-32	139	51.1
	>=33	31	11.4
Sex	Male	261	96.0
	Female	11	4.0
Driving experience	<1year	51	18.8
	1-5years	166	61.0
	6-10years	55	20.2
Level of education	No formal education	18	6.6
	Primary (1-8)	109	40.1
	Secondary (9-12)	90	33.1
	College or university	55	20.2
Marital status	Single	138	50.7
	Married	134	49.3

**First aid knowledge of motorcycle drivers**: Most participants, 191 (70.2%), defined first aid as immediate or initial care given to victims before trained medical personnel arrive, while 81 (29.8%) defined it as care given only by trained professionals. The necessity of providing first aid immediately at the scene was identified by 174 (64.0%). More than half, 152 (55.9%), believed first aid should be administered by trained bystanders.

Regarding airway management, 127 (46.7%) did not know how to open a blocked airway. Only 102 (37.5%) knew that priority should be given to MCA victims during first aid. Concerning management of severe bleeding, 248 (91.2%) responded correctly, identifying measures such as applying a tourniquet, tying the bleeding site with a cloth or bandage, applying pressure, and elevating the injured part. In contrast, only 18 (6.6%) knew safe positions for trauma victims, such as placing the victim on their side while stabilizing the neck and head or in a face-up position. A total of 174 (64%) participants were aware of immobilization using splints for managing MCA victims (Table 2). Overall, about 55% of participants demonstrated good knowledge of first aid.

**Table 2:** Frequency distribution of first aid knowledge of motorcycle drivers, Dilla town, southern Ethiopia

Variable	Response	Frequency	Percent
	Care given to victims by only trained medical professionals	81	29.8
Define first aid	Immediate or initial care given to victims before trained medical workers arrive	191	70.2
Timing of and place for first aid	Immediately at scene	174	64.0
	At health facility	92	33.8
	I don't know	6	2.2
	Health care workers only	119	43.8
Provider of first aid	Trained bystanders including drives	152	55.9
	I don't know	1	0.4
	Jaw thrust	88	32.54
Procedures to open air way	Head tilt and chin lift	57	21.05
	I don't know	127	46.75
Not component of the first aid management	Air way problem maintenance	38	14.0
	Stopping bleeding through applying pressure	21	7.7
	Splinting fracture	34	12.5
	Giving oral fluid for unconscious	107	39.3
	Transported the victim to hospital	5	1.8
	I don't know	66	24.3
	Airway problem maintenance	102	37.5
Prioritized/first done for victims	Transport to hospital	55	20.2
	
	Stopping bleeding	92	33.8
	Splinting fracture	6	2.2
	I don't know	17	6.3
	Unconscious	54	19.9
Sign of air way problem	No breathing	192	70.6
	Unconscious, no breathing	25	9.2
	I don't know	1	0.4
	Mouth to mouth	102	37.5
Ways to give breath	Mouth to nose	61	22.4
	I don't know	108	39.7
	Apply tourniquet	60	22.1
	Tying the bleeding site with close/bandage	141	51.8
	Apply pressure and lift the injured part above the body level	47	17.3
Important action to stop sever bleeding	Apply tourniquet and tying the bleeding site with close/bandage	10	3.7
	I don't know	14	5.1
	Placing the victim sideways	69	25.4
Safe position during traumatic event	Keeping the neck and head not to move and face up position	135	49.6
	Keeping the patient face down position	18	6.6
	Placing the victim sideways and keeping the neck and head not to move and face up position	18	6.6
	I don't know	32	11.8
	Immobilization using splinting	174	64.0
Management of the bone fracture	Try twist back into the place by force	54	19.9
	I don't know	44	16.2

**First aid attitude of motorcycle drivers**: Most respondents, 220 (80.9%), believed first aid should be given immediately at the scene. In addition, 198 (72.2%) expressed willingness to provide first aid whenever an accident occurs. Among those unwilling to provide first aid, reasons included fear of applying the wrong treatment and causing harm (32; 11.8%), fear of infection (15; 5.5%), lack of skills (26; 9.6%), and overcrowding at the scene (1; 0.4%). Conversely, 254 (93.4%) participants expressed interest in receiving first aid training. However, 70 (25.7%) believed training should not be provided to non-professionals (Table 3). In total, 140 (51.5%) demonstrated a positive attitude toward first aid, while 132 (48.5%) had a negative attitude.

**Table 3:** Frequency distribution of first aid attitude and practice of motorcycle drivers, Dilla, southern, Ethiopia, 2023

A. First aid attitude of motorcycle drivers			

Variable	Response	Frequency	Percent
Believe first aid immediately at scene	Yes	220	80.9
	No	37	13.6
	Uncertain	15	5.5
Willingness to provide first aid	Yes	198	72.8
	No	74	27.2
Interest in training first aid	Yes	254	93.4
	No	18	6.6
Neck injury aggravated by moving	Yes	114	41.9
	No	51	18.8
	Uncertain	107	39.3
Lay people training	Yes	151	55.5
	No	70	25.7
	I don't know	51	18.8

B. First aid attitude and practice of motorcycle drivers			
Attended to MCA victims	Yes	259	95.2
	No	13	4.8
Accident occurs by whom.	by respondent	89	34.36
(n=259)	by another driver	170	65.64
Giving first aid (n=259)	Yes	109	42.1
	No	150	57.9
If no, why	I am not trained	27	18
(n=150)	Someone else did it	65	43.33
	Crowdedness of the incident site	33	22
	Frightened of doing harm than help	25	16.67
If yes, your action (n=109)	Call to ambulance	17	15.6
	Transfer to near hospital	41	37.61
	Give first aid for victims	51	46.79
Attended airway problem	Yes	78	30.1
	No	181	69.9
If yes, your action (n=78)	Call to ambulance	17	21.8
	Transfer to near hospital	34	43.59
	Give first aid and manage airway problems	27	34.61
Attended head and neck injury	Yes	171	66.02
	No	88	33.98
If yes, your first action (n=171)	keep the neck and head does not move and transfer to nearest hospital	77	45.02
	Call to ambulance	87	50.88
	Call to ambulance and keep the neck and head does not move and transfer to nearest hospital	3	1.76
Attended severe bleeding	Yes	168	64.87
	No	91	35.13
If yes, your first action	Apply pressure and dress	62	36.9
	Apply tourniquet	15	8.92
	Call ambulance	10	5.95
	Transfer to hospital	55	32.72
Attended bone fracture	Yes	97	37.45
	No	162	62.55
If yes, your first action (n=97)	Call to ambulance	39	40.20
	Immobilization using splinting	51	52.58
Attended other than above mentioned	Yes	7	2.7
	No	252	97.3
If yes, type of injury (n=7)	Broken teeth	1	14.29
	Burning of leg in gas outlet	2	28.57
	Death	2	28.57
	Nasal bleeding	1	14.29
	Swelling of lower extremity	1	14.29
	Add coffee powder on it	1	14.3
If yes, your first action	Cold compress	1	14.3
	Nothing	1	14.3
	Pinch the nose	1	14.3
	Transport to hospital	3	42.8

**First aid practice of motorcycle drivers**: Almost all respondents, 259 (95.2%), witnessed an accident scene. Among them, 89 (34.36%) were involved in the accident themselves, while 170 (65.64%) witnessed accidents involving other motorcycle drivers. Only 109 (42.1%) reported giving first aid. Reasons for not giving first aid included another person already providing it (65; 43.33%), overcrowding at the scene (33; 22%), lack of training (27; 18%), and fear of causing harm (25; 16.67%).

Of the 78 (30.1%) who witnessed an airway problem, 34 (43.59%) transported the victim to a hospital. Among 97 (37.45%) who observed a bone fracture, 51 (52.58%) reported immobilization using splints as their first action. A small proportion (7; 2.7%) encountered other injuries, such as death, burns to the lower extremities, and broken teeth. Of these, three (42.86%) reported transporting the victim to a hospital as their first action.

Among the 168 (64.87%) who attended to severe bleeding, first responses included applying pressure and dressing (62; 36.9%), transporting to a nearby hospital (55; 32.72%), applying a tourniquet (15; 8.92%), and calling an ambulance (10; 5.95%) (Table 3). Overall, 109 (40.1%) demonstrated good practice, while 163 (59.9%) showed poor practice.

**Factors associated with first aid knowledge**: In binary logistic regression, age, marital status, driving experience, and education were associated with knowledge (p < 0.25). In multivariate logistic regression, only education remained significant. Respondents with college or university education (AOR = 3.937; 95% CI: 1.175–13.194) and those with secondary education (AOR = 3.40; 95% CI: 1.1–10.3) were about four times more likely to have good knowledge compared to uneducated respondents (Table 4).

**Table 4:** Factors associated to first aid knowledge, attitude and practice among motorcycle drivers, Dilla, southern Ethiopia, 2023

A. Factors associated to first aid knowledge among motorcycle drivers						
Factors		Knowledge		COR (95%CI)	AOR (95%CI)	P-value

		Poor	Good			
Age	17-24	62(60.8%)	40(39.2%)	1		
	25-32	50(36%)	89(64%)	2.76(1.6-4.67)	1.9(0.96-3.96)	0.064
	>=33	11(35.5%)	20(64.5%)	2.81(1.2-6.5)	1.98(0.61-6.4)	0.256
Marital status	Single	76(55.07%)	62(44.93%)	1		
	Married	47(35.07%)	87(64.93%)	2.269(1.39-3.69)	1.39(0.6-2.89)	0.373
Level of education	No education	12(66.66%)	6(33.34%)	1		
	Primary education	60(55.04%)	49(44.96%)	1.63(.571-4.66)	2.11(0.69-6.4)	0.190
	Secondary education	35(38.9%)	55(61.1%)	3.14(1.081-9.14)	3.40(1.1-10.3)	0.031*
	College/university	16(29.1%)	39(70.9%)	4.87(1.56-15.23)	3.9(1.1-13.19)	0.026*
Driving experience	<1year	32(62.74%)	19(37.26%)	1		
	1-5 year	66(39.76%)	100(60.24%)	2.5(1.33-4.87)	1.61(0.7-3.28)	0.192
	6-10 year	25(45.45%)	30(54.55%)	2.02(0.93-4.4)	0.71(0.26-1.9)	0.508

**B. Factors associated with first aid attitude among motorcycle drivers**						

**Factors**		**Attitude**		**COR (95%CI)**	**AOR (95%CI)**	**P-value**

		**Negative**	**Positive**			

Age	17-24	61(59.8%)	41(40.2%)	1		
	25-32	60(43.17%)	79(56.83%)	1.95(1.16-3.29)	0.36(0.12-1.08)	0.070
	>=33	11(35.48%)	20(64.52%)	2.70(1.17-6.23)	0.71(0.30-1.68)	0.442
Marital status	Single	76(55.07%)	62(44.93%)	1		
	Married	56(41.8%)	78(58.2%)	1.70(1.05-2.75)	0.97(0.48-1.95)	0.941
Level of education	No education	6(33.33%)	12(66.67%)	1.43(0.47-4.39)	2.14(0.65-6.95)	0.206
	Primary education	61(56%)	48(44%)	0.56(0.29-1.09)	0.91(0.41-2.02)	0.817
	Secondary education	42(46.7%)	48(53.3%)	0.82(0.41-1.61)	1.08(0.52-2.25)	0.829
	College or university	23(41.8%)	32(58.2%)	1		

**C. Factors associated with first aid practice among motorcycle drivers**						

**Factors**		**Practice**		**COR (95%CI)**	**AOR (95%CI)**	**P-value**

		**Poor**	**Good**			

Age	17-24	71(26.1%)	31(11.4%)	1		
	25-32	80(29.4%)	59(21.7%)	1.68(0.98-2.89)	1.93(0.97-3.8)	0.058
	>=33	12(4.4%)	19(7.0%)	3.6(1.57-8.37)	4.4(1.56-12.6)	0.005*
Marital status	Single	89(32.7%)	49(18.0%)	1		
	Married	74(27.2%)	60(22.1%)	1.47(0.9-2.39)	0.8(0.41-1.57)	0.524

* significantly associated

**Factors associated with first aid attitude**: Age, marital status, and education were associated with attitudes in binary logistic regression and were included in the multivariate model. However, none showed significant associations in multivariate logistic regression (Table 4).

**Factors associated with first aid practice**: In binary logistic regression, age and marital status were associated with first aid practice. In multivariate analysis, only age was significant: respondents aged ≥33 years were four times more likely to practice first aid compared to those aged 17–24 years (AOR = 4.4; 95% CI: 1.56–12.64) (Table 4).

## Discussion

To our knowledge, no prior studies have assessed the knowledge, attitude, and practice of first aid among motorcycle drivers in Ethiopia. This study is the first to describe KAP toward first aid services among motorcycle drivers in Dilla town, Southern Ethiopia.

In this study, 96% of respondents were male, and more than half were aged 26–32 years, with a mean age of 26.21 (SD ±4.95). A study in Benin (15) reported all respondents were male, with an average age of 38.38 years, and more than three-fourths were aged 30–44. A Kenyan study (17) on boda-boda drivers reported a mean age of 29 years, with an age range of 17–59 years. The male dominance observed in these studies is linked to cultural norms that portray motorcycle driving as male-dominated. Compared with Benin and Kenya, respondents in our study were younger, likely because younger individuals use motorcycles as a source of income, reflecting regional differences in age distribution.

In our study, 54.8% of respondents had good knowledge, higher than findings from a Nigerian study (18), where only 2.3% had good knowledge. This discrepancy may be due to differences in assessment tools or sociodemographic factors. Education was significantly associated with knowledge in our study, consistent with the idea that individuals with higher education have more access to health information and better comprehension of first aid principles.

Knowledge of placing a victim in the lateral safety position was reported by 25.4% of respondents, similar to 21.6% in Benin^15^. Knowledge of how to open a blocked airway was reported by 32.5% of respondents, higher than the 4% reported in Kenya (17). Differences in awareness, access to health information, and exposure to trauma may explain this variation.

About 51.5% of participants in our study had a positive attitude toward first aid, with 72.2% willing to provide assistance. Among those unwilling, 9.6% cited lack of knowledge. This finding is comparable to Kenya (17), where 91% were willing to provide first aid, while 9% were not. Similarly, in Benin (15), 19.64% cited lack of knowledge as the reason for unwillingness. Differences in training exposure or real-life emergency experience may explain the variation in willingness to help.

Regarding practice, 95.2% of participants had attended accident scenes, but only 40.1% demonstrated good practice. Of those who attended accidents, 42.1% provided first aid, and 57.9% did not. Among those who did, 14% called an ambulance and 37.6% transferred victims to a hospital. In Benin (15), 32.27% reported providing first aid, while in Kenya (17), 73% primarily transported victims to hospitals, 14% called for help, and 6.5% gave first aid.

In this study, 34.36% reported being involved in accidents themselves, compared to 53% in Kenya. Variations may be due to differences in timing, accident frequency, road conditions, or road safety awareness. Age was significantly associated with first aid practice, with older drivers more likely to provide assistance, possibly due to greater experience and exposure.

This study was limited to registered motorcycles with plate numbers. Despite this, as the first study of its kind in Ethiopia, it addressed key variables. Further studies with larger sample sizes, including unregistered motorcycles, are recommended to expand knowledge on KAP toward first aid.

In conclusion, the study found that nearly half of motorcycle drivers in Dilla town had adequate knowledge and positive attitudes toward first aid, but only about two-fifths demonstrated good practice. Higer education level and older age were significantly associated with good knowledge and practice, respectively.
